# Thermo-optic tuning of silicon nitride microring resonators with low loss non-volatile $\hbox {Sb}_{2}\hbox {S}_{3}$ phase change material

**DOI:** 10.1038/s41598-022-21590-w

**Published:** 2022-10-24

**Authors:** Stefan T. Ilie, Joaquin Faneca, Ioannis Zeimpekis, Thalía Domínguez Bucio, Katarzyna Grabska, Daniel W. Hewak, Harold M. H. Chong, Frederic Y. Gardes

**Affiliations:** 1 Optoelectronics Research Centre, University of Southampton, Highfield, Southampton, SO17 1BJ UK; 2 Instituto de Microelectrónica de Barcelona, IMB-CNM (CSIC), Campus UAB, 08193 Bellaterra, Barcelona Spain; 3 School of Electronics and Computer Science, University of Southampton, Southampton, SO17 1BJ UK

**Keywords:** Materials science, Optics and photonics

## Abstract

A new family of phase change material based on antimony has recently been explored for applications in near-IR tunable photonics due to its wide bandgap, manifested as broadband transparency from visible to NIR wavelengths. Here, we characterize $\hbox {Sb}_{2} \hbox {S}_{3}$ optically and demonstrate the integration of this phase change material in a silicon nitride platform using a microring resonator that can be thermally tuned using the amorphous and crystalline states of the phase change material, achieving extinction ratios of up to 18 dB in the C-band. We extract the thermo-optic coefficient of the amorphous and crystalline states of the $\hbox {Sb}_{2}\hbox {S}_{3}$ to be 3.4 x $10^{-4}\hbox {K}^{-1}$ and 0.1 x 10$^{-4}\hbox {K}^{-1}$, respectively. Additionally, we detail the first observation of bi-directional shifting for permanent trimming of a non-volatile switch using continuous wave (CW) laser exposure ($-5.9$ to 5.1 dBm) with a modulation in effective refractive index ranging from +5.23 x $10^{-5}$ to $-1.20$ x 10$^{-4}$. This work experimentally verifies optical phase modifications and permanent trimming of $\hbox {Sb}_{2}\hbox {S}_{3}$, enabling potential applications such as optically controlled memories and weights for neuromorphic architecture and high density switch matrix using a multi-layer PECVD based photonic integrated circuit.

## Introduction

The use of photonic integrated circuits (PICs) has gained momentum in recent years with increased functionality enabling multiple application domains, ranging from datacom^1^, bio sciences^2^, avionics, artificial neural networks^3^, and quantum information processing^4,5^ when integrated with the mainstream technology of silicon photonics. When PICs are designed and optimised to satisfy one specific application, they are called application-specific photonic integrated circuits (ASPICs)^6^. Alternatively, programmable PICs^7^ are based on the idea that light in an integrated photonic circuit can be manipulated either by routing or coupling to satisfy more than one application in the same chip, reducing the specific performance but increasing the flexibility. With the growing need of emerging applications that require flexibility and re-configurability while providing low-cost and low-power consumption^8^, photonic field programmable gate arrays (PFPGA) are increasing in interest^9^. The intrinsic limitations of the SOI platform^10^ has lead to further development in compatible alternative platforms, capable of complementing the functionality of silicon, such as aluminium nitride (AlN), alumina, ($\hbox {Al}_{2}\hbox {O}_{3}$), silicon carbide (SiC), indium phosphide (InP) and silicon nitride (SiNx). Among these materials, SiNx based PICs are a complementary CMOS compatible technology, which exploits its wide transparency range (0.4$-7\mu \hbox {m}$)^11^, refractive index tunability between 1.7 and 3.1^12,13^, multi-layer compatibility^14^, low temperature sensitivity and high tolerance to fabrication errors^12,15,16^, which can enable a widening of the field of applications^17^. Thermo-optic phase shifters built on SOI and SiNx platforms have achieved device footprint below 350 $\mu$m but suffer from slow modulation speed and high power consumption^18,19^. Due to its insulating nature and centro-symmetric crystalline structure, optical modulation based on charge diffusion and respectively electro-optic effects cannot be applied directly when using SiNx^20^. However, optical phase modulation has been previously achieved by the integration of graphene^21,22^, $\hbox {LiNbO}_{{3}}$^23^, (EO) polymers^24,25^ and BTO^26^ on SiNx, but require complex fabrication schemes such as planarization and bonding and can suffer from degradation at high operating temperatures resulting in potential yield issues^27–29^.

Phase change materials (PCMs) have been investigated in the context of PICs due to their non-volatile phase transition and for the high refractive index contrast between their amorphous and crystalline phases^30–32^. The ability to reconfigure PICs has been implemented in applications ranging from memories^33^, wavelength division multiplexing^34^, and switches^30^ to neuromorphic devices^35–37^. Amongst all of the ternary phase change material in non-volatile integrated photonic systems, the most used is $\hbox {Ge}_{2}\hbox {Sb}_{2}\hbox {Te}_{5}$ (GST). Although it shows fast switching (ns) and stability in resistive switching applications,^38,39^ the performance of GST-based integrated photonics is limited by strong coupling between the amplitude and phase modulation caused by a metal-insulator transition (MIT)^40,41^, which induces excessive optical loss in the crystalline phase of the material. This makes it ideal for reconfigurable silicon photonic applications such as switches, filters, memories and matrix multiplication, to name a few,^30,31,42,43^ by providing energy-efficient, compact, non-volatile operation.

A PCM with a wide band-gap, low absorption in both amorphous and crystalline states can ease these limitations and provide a path towards non-volatile phase modulation with low-loss for large scale neuromorphic computing^44^ and optical field programmable gate arrays (OFPGAs)^45^. A new family of phase-change materials based on $\hbox {Sb}_{2}\hbox {S}_{3}$ and $\hbox {Sb}_{2}\hbox {Se}_{3}$^46,47^ has emerged as a promising candidate due to its low absorption in both states at $\lambda$=1.55 $\mu$m. $\hbox {Sb}_{{2}}\hbox {S}_{{3}}$ exhibits a broadband transparency ranging from 0.6 $\mu$m up to the near-IR, a refractive index contrast ($\Delta$n) between its states of 0.60 at 1.55 $\mu$m and low inherent losses with extinction coefficient *k* values lower than 10$^{-4}$ in both phases. Devices such as Bragg gratings^48^, Mach-Zehnder interferometers (MZIs)^49^, Multi-Mode interferometers (MMIs)^50^ and ring resonator (RR) modulators on SOI^51,52^ have recently been demonstrated using these materials.

**Figure 1:**
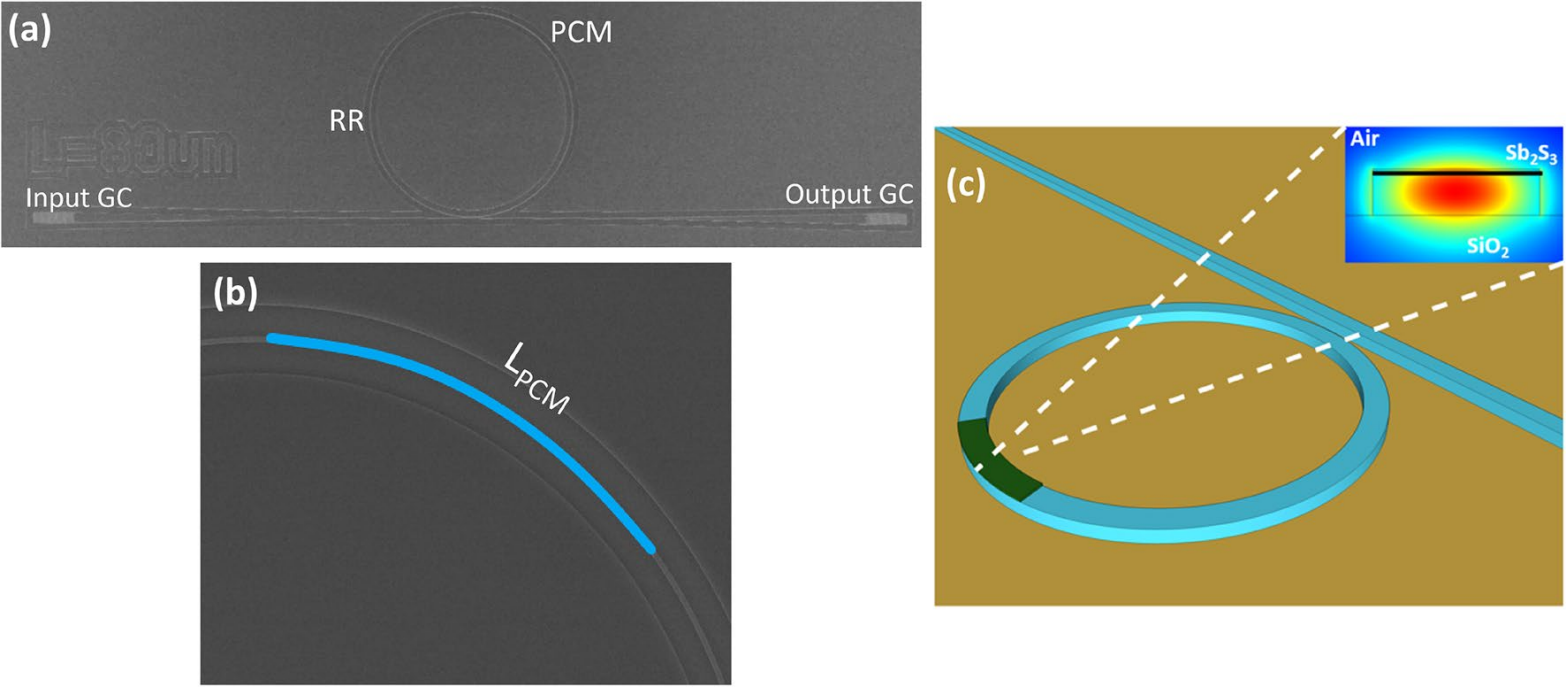
(**a**) SEM image of a RR design variation with the PCM cell deposited on top. (**b**) Zoom in SEM image of a PCM cell highlighted in blue colour (**c**) Schematic of SiNx microring resonator partially covered with $\hbox {Sb}_{2}\hbox {S}_{3}$, with an inset showing a cross-section of the RR and the fundamental TE optical mode.

In this work, we experimentally demonstrate the tuning of RR building blocks on a silicon nitride hybrid platform based on $\hbox {Sb}_{2}\hbox {S}_{3}$. The thermo-optic coefficient of the PCM was extracted experimentally at a wavelength of 1.55 $\mu \hbox {m}$ and was found to be 3.4 x 10$^{-4}\hbox {K}^{-1}$ in the amorphous state and 0.1 x 10$^{-4}\hbox {K}^{-1}$ in the crystalline state. This work demonstrates a method for permanent post fabrication trimming by tuning the optical properties of $\hbox {Sb}_{2} \hbox {S}_{3}$ cladded RRs at the telecom wavelengths. We report on the first observation of bi-directional shifting for permanent trimming of RRs using a crystallised PCM and a continuous wave (CW) laser at powers ranging from −5.9 to 5.1 dBm inside the waveguide, achieving a non-linear maximum resonant wavelength shift from −23 to +10 pm respectively, for a period of 60 minutes upon exposure in a RR with a 100 $\mu$m diameter and a $\hbox {Sb}_{{2}}\hbox {S}_{{3}}$ PCM cell of 120 $\mu$m.

## Fabrication and characterisation

Ring resonators were fabricated on 8” Si wafers with a 2 $\mu$m $\hbox {SiO}_{2}$ on top of which a 300 nm SiNx layer (n = 2.0) was deposited at 350$^{\circ }$C using the $\hbox {NH}_{{3}}$-free PECVD process detailed in^12^. The patterns were defined by means of 245 nm deep-UV (DUV) lithography and then 300 nm fully etched ridge waveguides were made by an inductively coupled plasma reactive ion etching (ICP-RIE) process. A secondary lithography step was used to create windows for the PCMs deposition, after which a 20 ± 5 nm layer of $\hbox {Sb}_{{2}}\hbox {S}_{{3}}$ was deposited using RF sputtering followed by the deposition of a 10 nm ZnS/$\hbox {SiO}_{2}$ layer to protect the PCM and prevent sulfur loss^47^. The PCM lift-off was completed by the sequential dipping of the samples in acetone and IPA at room temperature followed by NMP at 80 °C with ultrasonic agitation. Finally, a secondary 10 nm layer of 20−80% ZnS-$\hbox {SiO}_{2}$ was deposited over the PIC to provide additional protection to the exposed PCM sidewalls and to provide encapsulation to the PCM material edge during melt. The phase change of the material from amorphous to the crystalline state was thermally induced by heating the chip on a hot plate at the crystallisation temperature (270 $^{\circ }$C) for 10 minutes in an air filtered environment.

The spectral response of all the devices was characterised using a tunable laser source system (Agilent 8163B Lightwave Multimeter), followed by a C-band erbium doped laser amplifier (EAD-1K-C), an optical fiber attenuator (VOA50PM-APC), the device under test (DUT) and finally the InGaAs photo-detector. The polarization of the light was controlled to ensure that only TE modes could propagate through the devices and the measurements were normalized to extract the loss contribution of the input/output grating couplers and the waveguide. In order to extract the spectral response at different powers, the following methodology was used: Firstly, a 10 dBm pump-laser sweep was done before every single-wavelength laser exposure. Secondly, a 60 minute continuous exposure was done at a fixed wavelength within the resonant dip of the RRs. During the exposure, a photo-detector was used to monitor the response of the RR in real-time. Finally, a second low pump power (10 dBm) sweep was conducted for characterisation purposes, followed by a cool-down period of 5 minutes before the next high power fixed wavelength exposure cycle was performed on the same device. Two distinct pump-laser sweeps are done in order to monitor the position of the resonant wavelength of the RRs before and after each exposure.

## Results and discussion

### Sb_2_S_3_ on SiNx microring resonators

**Figure 2:**
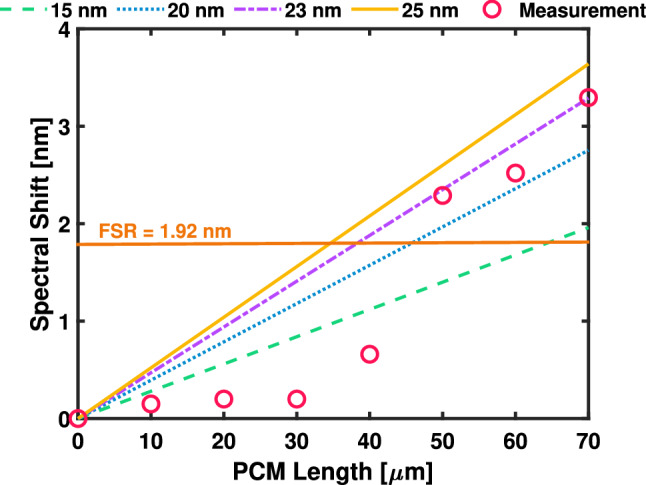
Simulated spectral shift from crystalline to amorphous for $\hbox {Sb2}_{{2}}\hbox {S}_{{3}}$-cell lengths ranging from 10 $\mu$m to 70 $\mu$m for a PCM thickness of 15 nm (green-dash line), 20 nm (blue-dotted line), 23 nm (purple dash-dot line), 25 nm (yellow-solid line) and optical measurement (red).

Different PCM lengths between 10 $\mu$m and 120 $\mu$m were deposited on identical SiNx microring resonators with a radius of 100 $\mu$m and a gap between the RR and the bus-waveguide of 0.25 $\mu$m to investigate the optical spectral shift. Figure 1 (a,b) shows SEM pictures of one of the RR variations alongside a zoomed in image of the PCM cell deposited on the top of the arc section. Figure 1c shows the schematic of the SiNx RR alongside with the cross-section for the fundamental TE optical mode. All the devices include input and output grating couplers (GCs) designed to couple light at −7.5° off the normal, which consist of a surface grating area of 10 $\mu$m x 37 $\mu$m and a grating period of 1.238 $\mu$m tapered down to a single-mode width of 1.2 $\mu$m. The measured normalised insertion loss of the two GCs and the waveguide was measured to be $28.23\,\pm \,0.42$ dB at $\lambda$ = 1.55 $\mu$m. The bare RR structure without phase change material was characterised and normalised with respect to the GCs. Afterwards, the responses of the RRs for different lengths of the PCM cell were characterised in both amorphous and crystalline states. To quantitatively describe the induced modulation effect, Fig. 2 shows the simulated spectral shift ($\Delta \lambda _{Crys-Amp}$) between the crystalline and the amorphous state for cell lengths from 10 to 70 $\mu$m at different PCM thickness of 15, 20, 23 and 25 nm. The spectral shift per PCM unit length is extracted to be linearly growing with a rate of 0.028 nm /$\mu$m (green-dash line), 0.039 nm /$\mu$m (blue-dotted line), 0.047 nm /$\mu$m (purple dash-dot line) and 0.052 nm /$\mu$m (yellow-solid line) for a PCM thickness of 15, 20, 23 and 25 nm respectively.

The RRs exhibits a free-spectral range (FSR) of 1.92 nm at $\lambda$ = 1.55 $\mu$m, enabling an effective $\pi$-phase shift for a 41.84 $\mu$m PCM length and a 2$\pi$-phase shift for a 47.73 $\mu$m PCM length, respectively. For PCM lengths smaller than 30 $\mu$m, the mode interaction is minimal due to the small optical confinement of the mode in the PCM-layer, resulting in spectral shifts of 0.15 nm for a 10 $\mu$m - long PCM cell and of 0.2 nm for a 20 $\mu$m - long PCM cell. As the cell length increases above 50 $\mu$m, the interaction increases and the mode expands towards a higher refractive index material ($\hbox {Sb}_{{2}}\hbox {S}_{{3}}$), increasing the spectral shift and closely matching the simulated spectral shift for a PCM cell thickness of 23 nm.

**Figure 3:**
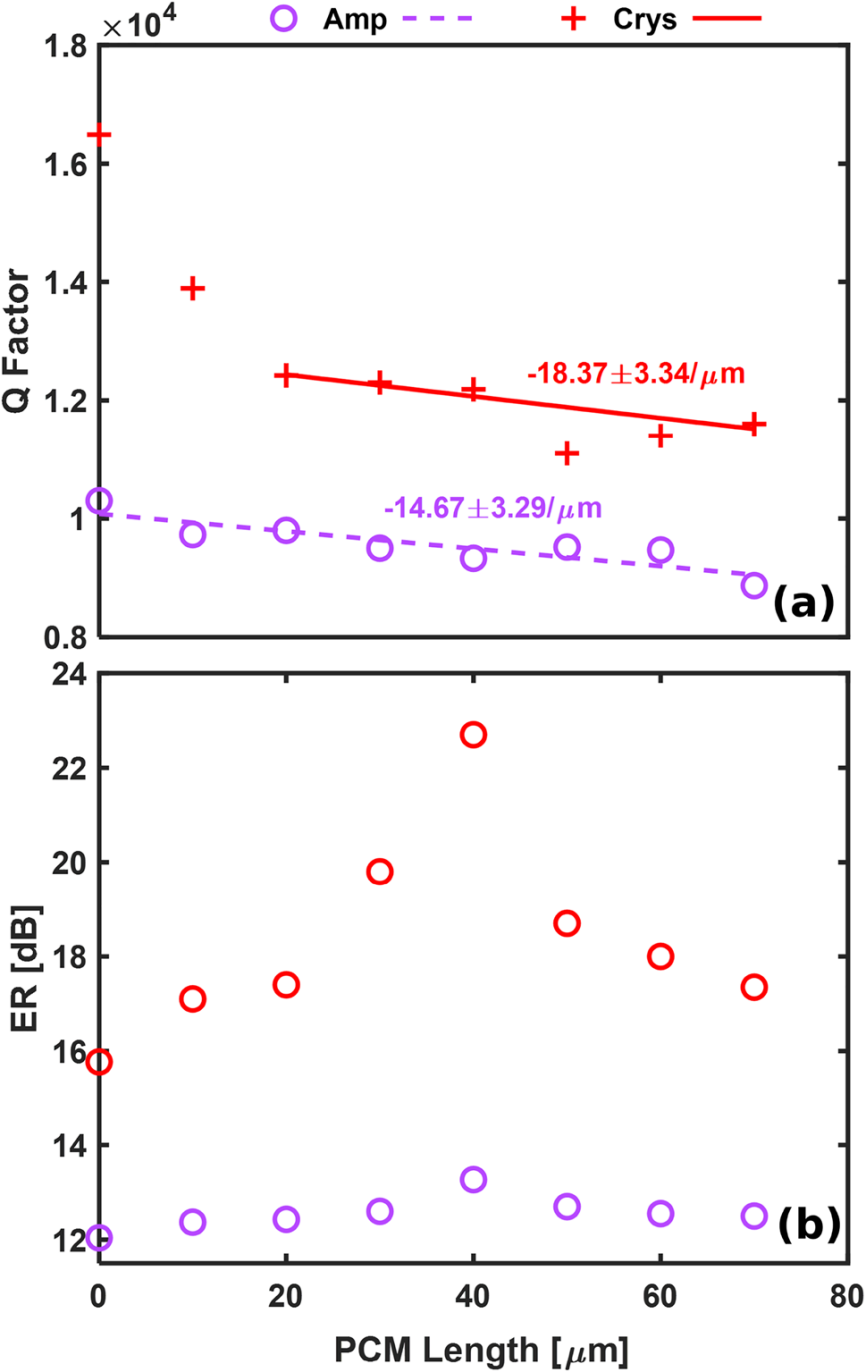
Measured $\hbox {Sb}_{{2}}\hbox {S}_{{3}}$ on SiN RR: (**a**) Q factor and (**b**) ER for PCM lengths ranging between 0 and 70 $\mu$m in amorphous (blue) and crystalline (red) states.

The resonance dip near 1.55 $\mu$m is fit with a Lorentzian function to extract the loaded quality factor (Q) (Fig. 3a) and the extinction ratio (ER) (Fig. 3b). In Fig. 3, a difference within the *Q* is produced when the PCM length is “zero” (bare RR) before and after the annealing process and we attribute this to the optical (refractive index) and electronic (sheet resistance) properties change induced by the annealing process (270 $^{\circ }$C) to the sputtered ZnS - $\hbox {SiO}_{{2}}$ protective layer^53,54^. The experimental data shows a linear fit for the Q-factors of the amorphous and crystalline states as the phase transition increases linearly with the length of $\hbox {Sb}_{{2}}\hbox {S}_{{3}}$. When considering the amorphous $\hbox {Sb}_{{2}}\hbox {S}_{{3}}$ (dashed line - R squared = 0.768) the Q-factor remains almost flat ($\Delta$Q/$\Delta \hbox {L}_{Sb_{2}S_{3}}$ = −14.67 ± 3.29 /$\mu$m), meaning that the impact associated with the PCM loss is very small on the response of the RRs. When considering the crystalline $\hbox {Sb}_{{2}}\hbox {S}_{{3}}$ (red line - R squared = 0.793), the Q factor decreases with a steeper rate ($\Delta$Q/$\Delta \hbox {L}_{Sb_{2}S_{3}}$ = $-18.37 \pm 3.34$ /$\mu$m) when compared to the amorphous $\hbox {Sb}_{{2}}\hbox {S}_{{3}}$, showing higher induced optical loss within the resonant cavity, as also confirmed by previously reported measurements^49^. The average ERs of the resonances in the amorphous state of the PCM is only of 12.5 dB, while in the crystalline state it is 18 dB over the wavelength range centered at $\lambda$ = 1.55 $\mu$m. The extinction ratio and the quality factor depends on the cladding refractive index. In the case of having air-cladding, the effective refractive index of the mode would be modified, affecting the optical properties of the RRs.

The maximum ER value of 22.715 dB is achieved for a $\hbox {L}_{Sb_{2}S_{3}}$ = 40 $\mu$m in the crystalline state and 13.275 dB in the amorphous state when critical coupling occurs. It is worth mentioning that the RRs were designed to be critically coupling for the crystalline state, hence the larger Q-factor and ER when the phase transition to the crystalline state occurs^51^. We note similar ER and Q-factor trends for both the amorphous and the crystalline state of $\hbox {Sb}_{{2}}\hbox {S}_{{3}}$ with regards to the higher optical insertion loss induced by longer PCM cells. The annealing process of the ZnS - $\hbox {SiO}_{{2}}$-layer associated with the crystallisation process has reduced the overall RR optical loss, which is shown by an improvement of the Q-factor and the ER of the bare SiNx RR by 60% and 30.9% respectively. The optical loss associated with the introduction of the $\hbox {Sb}_{{2}}\hbox {S}_{{3}}$-layer for either amorphous and the crystalline state is insignificant compared to the loss difference created by the ZnS - $\hbox {SiO}_{{2}}$-layer before and after the annealing process. We observe that the extinction ratio of the designed photonic switch is 9.425 dB when the critical coupling condition is satisfied for a fixed gap of 0.25 $\mu$m. This makes $\hbox {Sb}_{{2}}\hbox {S}_{{3}}$ cladded RRs very suitable for routing and filtering photonic systems due to its ability to maintain its ER over different PCM lengths and PCM states compared to GST cladded devices^31,42^, where the RRs resonances in the crystalline state are cancelled due to the PCM losses^55^.

### Thermo-optic effect of $\hbox {Sb}_{{2}}\hbox {S}_{{3}}$

**Figure 4:**
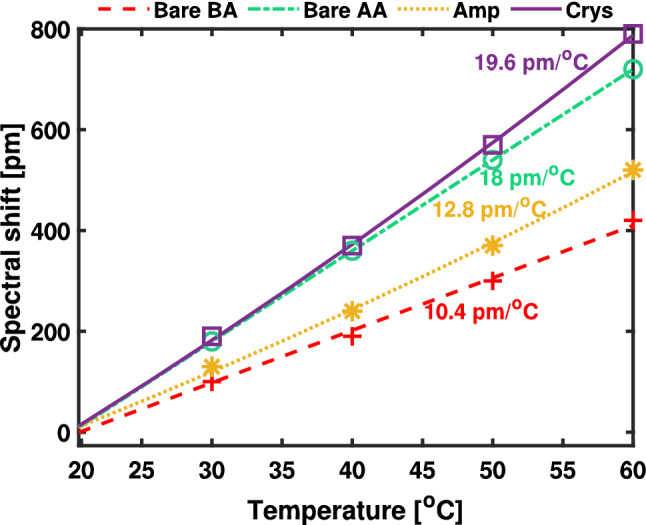
Experimental RR spectral shift for different temperatures, as indicated in the legends of: (**a**) Bare SiN RR before annealing (BA) (**b**) Bare SiN RR after annealing (AA) (**c**) Amp - $\hbox {Sb}_{{2}}\hbox {S}_{{3}}$ (**d**) Crys - $\hbox {Sb}_{{2}}\hbox {S}_{{3}}$ state at 1550 nm.

In this section, we investigate the thermo-optical (TO) effect of $\hbox {Sb}_{{2}}\hbox {S}_{{3}}$ in the C-band in the context of emerging high-density photonic applications. The TO characterisation allows to understand how temperature drift will affect the material and resulting optical phase depending on the crystallinity of the material. In our case, due to a setup limitation, the temperature dependence of the RR resonance is studied from 20 to 60 $^{\circ }$C using a thermal Peltier stage. To ensure heat distribution and thermal stability, each point in the measurement was taken after 30 min of setting the temperature. By adjusting the temperature of the thermoelectric element beneath the chip, the resonance wavelength of the RR could be tuned. Due to the crystallisation process and the associated optical changes within the ZnS - $\hbox {SiO}_{{2}}$ cladding layer (Sect. “Sb_2_S_3_ on SiNx microring resonators”), the wavelength sensitivity of a bare SiNx RR to temperature variations ($\frac{d\Lambda _{R}}{dT}$) changes between 10.8 pm/$^{\circ }$C before annealing to 18.0 pm/$^{\circ }$C after annealing (red and green lines in Fig. 4). Resonant wavelength changes of $\frac{d\Lambda _{R}}{dT}$ = 12.8 pm/$^{\circ }$C (2 pm/$^{\circ }$C bigger than the bare SiNx RR) were observed for the amorphous state (yellow dotted line) of the PCM with respect to the reference resonant wavelength at 20 °C temperature. The crystalline state (purple continuous line) exhibits shifts in resonant wavelength at a rate of $\frac{d\Lambda _{R}}{dT}$ = 19.6 pm/$^{\circ }$C (1.6 pm/$^{\circ }$C bigger than the bare SiN after annealing). Even though the ZnS - $\hbox {SiO}_{{2}}$-layer undergoes significant change in it’s thermo-optical properties once the devices have been annealed at the crystallisation temperature, we conclude that the optical losses to be similar to air-cladded devices^56^.

Through the commercial INTERCONNECT package of Lumerical Solutions, transmission models for the normalised devices are set up. The model consists of primary elements within the INTERCONNECT library and imported MODE profiles of cross-sections within the RR. Specifically, for the RR coupling section, the bidirectional directional coupler element was used, that takes as inputs the cross-coupling coefficients (*k* & *t*). An attenuator was used to adjust of the propagation loss in the waveguide and create a resonant mode in the RR cavity. The waveguide, bent waveguide and PCM section elements contains the simulated fundamental TE mode profile in the specific waveguide cross-section. Within the modelling of the dispersion and mode profile, ellipsometry values have been used for the SiNx, $\hbox {SiO}_{{2}}$, ZnS-$\hbox {SiO}_{{2}}$ and $\hbox {Sb}_{{2}}\hbox {S}_{{3}}$ at the wavelength range of interest (1540-1560 nm). The fitted values of the TO coefficient are indicative of the order of magnitude of change in response of the RR with regards to a temperature change. The fit of the spectra along the calculated TO coefficient of the SiN-$\hbox {Sb}_{{2}}\hbox {S}_{{3}}$ RR at $\lambda$ = $1.55\mu \hbox {m}$ is presented in Table 1, in which BA refers to the TO coefficient of ZnS-$\hbox {SiO}_{{2}}$ before annealing, AA, refers to the TO coefficient of the ZnS-$\hbox {SiO}_{{2}}$ after annealing, AMP and CRYS refers to the $\hbox {Sb}_{{2}}\hbox {S}_{{3}}$ in amorphous and crystalline states respectively. The TO coefficient of the materials are a good fit with regards to the experimental data using a first-order polynomial equation with a standard error of ± 0.37 pm/°C for the ZnS-$\hbox {SiO}_{{2}}$ before annealing, 0 for the ZnS-$\hbox {SiO}_{{2}}$ after annealing, ± 0.32 pm/°C for the amorphous $\hbox {Sb}_{{2}}\hbox {S}_{{3}}$ and ± 0.32 pm/°C for the crystalline $\hbox {Sb}_{{2}}\hbox {S}_{{3}}$. We use the relative position of the resonant shifts with regards to the reference ($\Delta$T = 0) based on 4 temperatures sets at ($\Delta$T = 10, 20, 30, and 40 $^{0}$C). The TO coefficient of the ZnS-$\hbox {SiO}_{{2}}$ before and after annealing was fit using a SiNx RR coated with ZnS-$\hbox {SiO}_{{2}}$. The TO coefficient of the amorphous and crystalline $\hbox {Sb}_{{2}}\hbox {S}_{{3}}$ was fit using the calculated ZnS-$\hbox {SiO}_{{2}}$ TO coefficients.

The TO coefficient of $\hbox {SiO}_{{2}}$^57^ is approximately 1.00 x 10$^{-5} \hbox {K}^{-1}$, while the TO coefficient of the deposited stoichiometric $\hbox {NH}_{{3}}$-PECVD SiNx-films^58^ is 1.00 x 10$^{-5} \hbox {K}^{-1}$. The TO coefficient for ZnS-$\hbox {SiO}_{{2}}$ is fit to be 2.35 x 10$^{-4}$ and 9.375 x 10$^{-4}$ before annealing and after annealing respectively. While the TO coefficient of the amorphous and crystalline $\hbox {Sb}_{{2}}\hbox {S}_{{3}}$ are 3.8± 0.9 x 10$^{-4} \hbox {K}^{-1}$ and 0.1 x 10$^{-4} \hbox {K}^{-1}$, given a PCM thickness of 20 nm and a variation of ± 5 nm. The fitted TO for a PCM thickness of 23 nm is 3.4 x 10$^{-4} \hbox {K}^{-1}$. We note that the ZnS-$\hbox {SiO}_{{2}}$ cladding dominates the TO of the crystalline state of the PCM (two order of magnitude difference), which results in the ZnS-$\hbox {SiO}_{{2}}$ greatly dominating the optical resonant shift rate compared to the PCM, irrespective of the PCM thickness variation. However, in the amorphous state, both TO coefficients of the ZnS-$\hbox {SiO}_{{2}}$ cladding and the PCM are comparable and have similar influence in the observed optical shift. When the $\hbox {Sb}_{{2}}\hbox {S}_{{3}}$ PCM layer is deposited on top of a silicon waveguiding platform, the PCM TO coefficients obtained are −3.11 x 10$^{-4} \hbox {K}^{-1}$ and −7.28 x 10$^{-4} \hbox {K}^{-1}$ for the amorphous and crystalline states^51^ respectively. A similarly low TO coefficient is observed for the crystalline $\hbox {Sb}_{{2}}\hbox {S}_{{3}}$ when deposited on a SiNx-platform, compared with the amorphous one. Albeit the difference, the trend is the same, in which the TO coefficient of the amorphous $\hbox {Sb}_{{2}}\hbox {S}_{{3}}$ is bigger than that of the crystalline $\hbox {Sb}_{{2}}\hbox {S}_{{3}}$. We attribute this to the different deposition method (DC sputtered compared to RF sputtering) and local changes in the film stoichiometry^59^. Increasing the PCM thickness, would result in increased mode mismatch. Furthermore, due to the fact the $\hbox {Sb}_{{2}}\hbox {S}_{{3}}$ has a bigger refractive index than $\hbox {SiN}_{{x}}$, thicker PCM layers would allow the PCM to support optical modes, resulting in high optical absorption. Thin PCM-layers have been previously used to facilitate thermal diffusion required for phase-cycling^60^. Similarly, we chose a thin-layer of 10 nm ZnS-$\hbox {SiO}_{{2}}$ because the grating couplers were optimised with air-cladding and having a thicker layer of ZnS-$\hbox {SiO}_{{2}}$ cladding will affect the grating performance. When grating couplers are designed for a thicker ZnS-$\hbox {SiO}_{{2}}$-layer, the stability of the PCM could improve^47^.

**Table 1:** Fitting summary of experimental data.

Material type	BA	AA	AMP	CRYS
Fit [pm/°C]	10.4	18	12.7	19.5
Standard Error [pm/°C]	± 0.37	0	± 0.32	± 0.32
Fitted TO [10$^{-4}\hbox {K}^{-1}$]	2.35	9.375	3.4	0.1

### Optically-induced trimming effect of $\hbox {Sb}_{{2}}\hbox {S}_{{3}}$

$\hbox {Sb}_{{2}}\hbox {S}_{{3}}$ has a significantly lower absorption loss in visible and near-infrared wavebands when compared to other PCMs (GST, GSST, GeTe or $\hbox {VO}_{{2}}$)^49,51,61–63^. Based on previous demonstrations, the as-deposited amorphous state PCM can be transformed to crystalline state by thermal annealing, which is accompanied with a strong change in optical properties. Moving towards the goal of achieving an application-specific photonic integrated circuit (ASPIC), Kun Gao et al.^64^ have demonstrated multi-level reversible and repeatable switching between the states of $\hbox {Sb}_{{2}}\hbox {S}_{{3}}$- by using femtosecond lasers, demonstrating the potential of $\hbox {Sb}_{{2}}\hbox {S}_{{3}}$-based tunable photonic devices. However, these methods rely on out-of-plane laser exposure exploiting the $\hbox {Sb}_{{2}}\hbox {S}_{{3}}$ bandgap region centered around 1.98 eV ($\lambda$ = 663-708 nm). If the $\hbox {Sb}_{{2}}\hbox {S}_{{3}}$ layer absorbs enough energy to generate heat, it will result in material alteration.

In this section, we investigate the permanent trimming^57^ (non-volatile tuning) of RRs integrated with a crystalline $\hbox {Sb}_{2} \hbox {S}_{{3}}$ cell using a C-band in-plane CW laser by tuning the resonant wavelength at low pump powers: 10, 12.55 and 14 dBm and high powers: 17 and 21 dBm. In order to estimate the power applied to the material in the waveguide, a loss of $14.1\,\pm \,0.21$ dB per GC is taken into account and an additional loss of $1.8\,\pm \,0.15$ dB was experimentally measured for the introduction of a power attenuator in the system, resulting in an optical waveguide power level of −5.9, −3.35, $-1.9 \,\pm \, 0.36$ dBm for the low pump powers, and 1.1, $5.1\,\pm \,0.36$ dBm for the high pump powers. The sample is annealed by hot-plate at 270 $^{\circ }$C for 10 min to crystallize the 120 $\mu$m long $\hbox {Sb}_{{2}}\hbox {S}_{{3}}$ cell as described in (Sect. "Fabrication and characterisation"), which increases the optical absorption by an estimated $\approx$ 3.7 dB^49^. The RR exhibits a FSR of 1.92 nm centred at $\lambda$ = 1.55 $\mu$m. Reference measurements were undertaken on the bare-SiN rings encapsulated with the ZnS-$\hbox {SiO}_{{2}}$-protective layer and no resonant wavelength shifts were observed when using waveguide optical powers within the range of −5.9 to 5.1 dBm.

**Figure 5:**
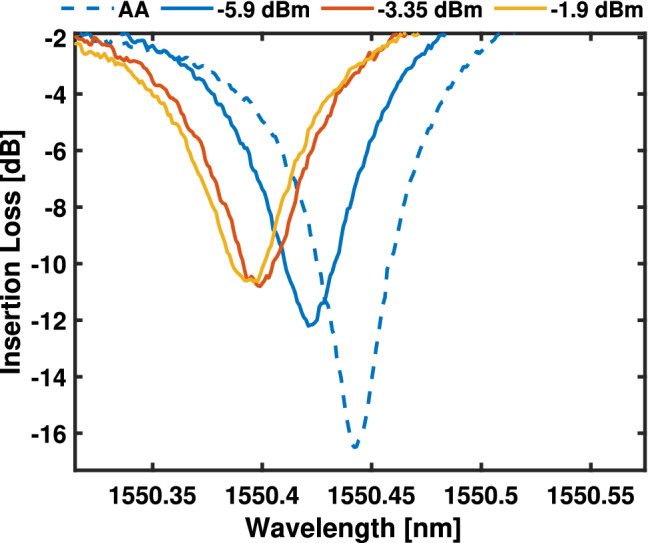
Normalised transmission of a RR AA (after annealing) partially capped with a crystalline $\hbox {Sb}_{{2}}\hbox {S}_{{3}}$ cell with a length of 120 $\mu$m using different optical powers in the waveguide (−5.9, −3.35 and −1.9 dBm) for a duration of 60 minutes.

To characterise the permanent trimming of the RRs, low-power (−5.9 dBm waveguide optical power) wavelength sweeps were done before and after laser exposure. When introducing an optical probe in the system, a portion of the optical power is absorbed and converted into heat. For a low-power probe, it is possible to read the transmission state of the $\hbox {Sb}_{{2}}\hbox {S}_{{3}}$ in a manner that does not influence the physical state of the material^33,65^. Figure 5 depicts the resonant wavelength change after exposure for a period of 60 minutes with a CW laser emitting around the resonant wavelength of the RR. After each exposure, the emitted wavelength was changed tracking the RR resonance ($\lambda$ = 1550.40, 1550.38 and 1550.37 nm − k = 0) for different waveguide optical powers (−5.9, −3.35 and −1.9 dBm). Figure 5 shows the initial −5.9 dBm power sweep and the resonant wavelength changes after the exposures under different powers of −5.9, −3.35 and −1.9 dBm for a duration of 60 minutes. To characterise the change in the shift rate over time and the effective refractive index modulation of the $\hbox {Sb}_{{2}}\hbox {S}_{{3}}$, a total of 5 wavelength sweeps were taken at different 15 minutes intervals (0, 15, 30, 45, 60 min). When considering a waveguide optical power of −5.9 dBm, a blue shift of $\Delta \lambda$ = −8, −12, −12, −13 pm with regards to the resonant wavelength under no laser exposure (after the thermal annealing of the PCM) is observed. For −3.35 dBm the $\Delta \lambda$ was −9, − 15, −18, −23 pm and lastly for −1.9 dBm the $\Delta \lambda$ was −11, −14, −15, −18 pm. This blue shift in the resonant wavelength is attributed to the system being exposed to laser heating with power densities below the melting threshold of the PCM that can activate a variety of temperature dependent processes within the solid material.^66,67^. The high temperatures generated enhance diffusion rates promoting impurity doping such as sulfur^68–70^ and the reorganization of the crystal structure, resulting in a temperature-dependant change in the optical properties of $\hbox {Sb}_{{2}}\hbox {S}_{{3}}$^71^.We speculate that the effect of crystal structure reorganisation and mobility of sulfur in $\hbox {Sb}_{{2}}\hbox {S}_{{3}}$, results in stronger changes than what it can be induced by a change of the capping layer contribution.

**Figure 6:**
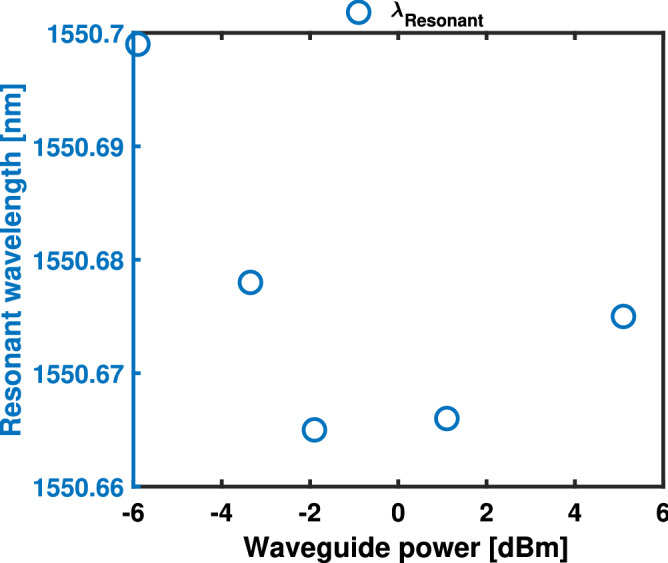
Resonant wavelength of the exposed RR against the injected optical power in the waveguide (−5.9–5.1 dBm) for a duration of 60 minutes, cladded with a crystalline 120 $\mu$m long $\hbox {Sb}_{{2}}\hbox {S}_{{3}}$ cell.

**Figure 7:**
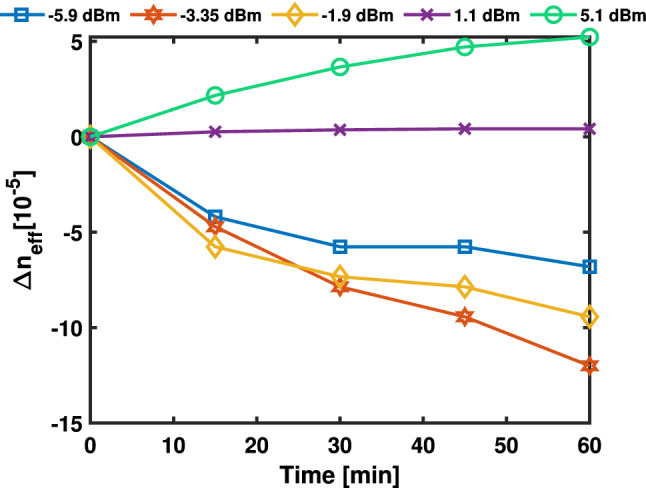
Extracted $\Delta n_{eff}$ of $\hbox {Sb}_{{2}}\hbox {S}_{{3}}$ in the crystalline state before and after each low-power exposure, as indicated in the legend (eye-guiding only).

To fully understand the non-volatile behaviour, high power exposures were undertaken using an erbium doped fiber amplifier (EDFA) coupled to a standard Fiber-DUT-Fiber optical transmission setup to obtain optical powers above −1.9 dBm inside the waveguide (Fig. 6), as detailed in (Sect. "Fabrication and characterisation"). When using optical powers between −1.9 and 1.1 dBm, a saturated non-linear effect is observed with negligible $\Delta \lambda$. While exposing the PCM at an optical waveguide power of 1.1 dBm a $\Delta \lambda$ = 0.5, 0.7, 0.8, 0.8 pm in the resonant wavelength change is observed when exposing the RR for 15, 30, 45 and 60 minutes, while for an optical waveguide power of 5.1 dBm, the $\Delta \lambda$ was 6, 8, 9, 10 pm. This red shift in the resonant wavelength is attributed to the system reaching the absorbed energy threshold within the $\hbox {Sb}_{{2}}\hbox {S}_{{3}}$ film to trigger a further partial crystallization. Once the PCM-cell has been exposed with a specific power, a thermo-dynamic equilibrium state is achieved in which no changes are observed when exposing with powers smaller than the applied power. Using the resonance shift and the applied power change, the effective refractive index modulation of the material $\Delta n_{eff}$ of SiN-$\hbox {Sb}_{{2}}\hbox {S}_{{3}}$ can be derived from the resonant condition of the RR^51,72^ as follows:

1 $$\begin{aligned} \Delta n_{eff} = \left( \frac{\lambda _{res}}{\lambda _{res0}}-1\right) \left( \frac{2\pi R - L_{Sb_{2}S_{3}}}{L_{Sb_{2}S_{3}}}n^{SiN}_{eff} + n_{eff0}\right) \end{aligned}$$

where $\Delta n_{eff}$ is the variation produced in the effective refractive index when the PCM cell is under the effect of the laser exposure when compared to the PCM in the crystalline state, $\lambda _{res0}$ and $\lambda _{res}$ are the resonant wavelengths before and after the applied optical power, $\hbox {n}_{{eff0}}$ and $\hbox {n}^{SiN}_{eff}$ are the effective refractive indexes of the $\hbox {Sb}_{{2}}\hbox {S}_{{3}}$-SiN hybrid waveguide mode and the bare SiN-waveguide respectively and $\hbox {L}_{Sb_{2}S_{3}}$ is the cell length. The modulation efficiency for different exposure powers is presented in Fig. 7. The effective refractive index contrast between the laser exposed PCM in crystalline states after 60 minutes was measured to be −6.80 x 10$^{-5}$, −1.20 x 10$^{-4}$, −9.42 x 10$^{-5}$, 4.19 x 10$^{-6}$ and 5.23 x 10$^{-5}$ for optical powers in the waveguide of −5.9, −3.35, −1.9, 1.1 and 5.1 dBm respectively. The volatile and non-volatile behaviour of PCMs such as GST on an integrated platform has been previously explored under CW laser exposure^65^. In this section, we demonstrated a passive permanent trimming method using only a continuous power optical probe without the interaction of optical pulse trains to assist in the phase transition exploiting the Sb_2_S_3_ in the crystalline state.

## Conclusion

In this paper, we demonstrate RRs building blocks exploiting an emerging family of low loss phase change materials in the C telecommunications band paving the way for future non-volatile electro-refractive modulation in mid index waveguides. RRs exhibiting a FSR of 1.92 nm, ERs > 20 dB and the ability to tune the whole FSR of a RR with a PCM cell length < 50 $\mu$m while switching between its amorphous and crystalline states are demonstrated. We extract the thermo-optic coefficient of amorphous and crystalline $\hbox {Sb}_{{2}}\hbox {S}_{{3}}$ to be 3.4 x 10$^{-4}\hbox {K}^{-1}$ and 0.1 x 10$^{-4}\hbox {K}^{-1}$ respectively. Additionally, we detailed the first observation of bi-directional shifting for the permanent trimming of a non-volatile switch using low-power CW laser exposure at 1.55 $\mu$m with the modulation of the effective refractive index ranging from +5.23 x 10$^{-5}$ to −1.20 x 10$^{-4}$ when using optical powers in the waveguide ranging from −5.9 to 5.1 dBm. This work demonstrates further insight for $\hbox {Sb}_{{2}}\hbox {S}_{{3}}$ integration in neuromorphic systems, scalable photonic integrated circuits and architectures for computing and routing applications.

## Data Availability

The data that support the findings of this study are available from the corresponding authors upon reasonable request.
